# The Key Role of Ambulatory Blood Pressure Monitoring in the Detection of Masked Hypertension and Other Phenomena in Frail Geriatric Patients

**DOI:** 10.3390/medicina57111221

**Published:** 2021-11-09

**Authors:** Marek Koudelka, Eliška Sovová

**Affiliations:** 1 Department of Exercise Medicine and Cardiovascular Rehabilitation, Palacký University Olomouc and University Hospital Olomouc, 77520 Olomouc, Czech Republic; eliska.sovova@fnol.cz; 2 Department of Internal Medicine, FRANZISKUS SPITAL GmbH, Landstraßer Hauptstraße 4a, 1030 Vienna, Austria

**Keywords:** ambulatory blood pressure monitoring (ABMP), frailty, geriatric patient, masked hypertension, masked uncontrolled hypertension

## Abstract

*Background and Objectives*: This study aims to determine prevalence of masked uncontrolled hypertension (MUH) in frail geriatric patients with arterial hypertension and thus show the role of ambulatory blood pressure monitoring (ABPM) since hypertension occurs in more than 80% of people 60+ years and cardiovascular diseases are the main cause of death worldwide. Despite modern pharmacotherapy, use of combination therapy and normal office blood pressure (BP), patients’ prognoses might worsen due to inadequate therapy (never-detected MUH). *Materials and Methods*: 118 frail geriatric patients (84.2 ± 4.4 years) treated for arterial hypertension with office BP < 140/90 mmHg participated in the study. 24-h ABPM and clinical examination were performed. *Results*: Although patients were normotensive in the office, 24-h measurements showed that BP values in 72% of hypertensives were not in the target range: MUH was identified in 47 (40%) patients during 24 h, in 48 (41%) patients during daytime and nocturnal hypertension in 60 (51%) patients. *Conclusions*: ABPM is essential for frail geriatric patients due to high prevalence of MUH, which cannot be detected based on office BP measurements. ABPM also helps to detect exaggerated morning surge, isolated systolic hypertension, dipping/non-dipping, and set and properly manage adequate treatment, which reduces incidence of cardiovascular events and contributes to decreasing the financial burden of society.

## 1. Introduction

High blood pressure (BP) remains the leading cause of morbidity and mortality worldwide. Every year, 7.6 million people die from hypertension, more than half of them are 45–69 years old [1]. It is alarming that in an ever-increasing group of people over 60, hypertension occurs in more than 80% of individuals [2]. In patients older than 80 years, the prevalence can be as high as 90% [3]. However, BP values increasing with age cannot be considered a normal and benign manifestation of aging. People who have normal BP in old age have the best cardiovascular (CV) prognosis. The risk group represents especially geriatric patients with very low or hardly any functional reserves (we speak of frail seniors). Geriatric frailty is generally reported in 10% of individuals aged ≥65 years and in 25–30% of individuals aged ≥85 years [4]. It significantly affects the patient’s prognosis and adjustment of medication is required to reduce the risk of fatal and non-fatal CV or cerebrovascular complications. Adverse drug effects and consequences of incorrect prescription are the most common causes of health alteration and hospitalization of geriatric patients. Rational and individualized pharmacotherapy is therefore an important part of geriatric patient care.

Essential hypertension and more severe target organ damage are more common in elderly patients than in the younger population. Hypertension occurs in individuals with a higher prevalence of coronary heart disease, myocardial infarction, diastolic dysfunction, a tendency to arrhythmia, and cerebral arteriosclerosis and peripheral arterial disease in general. Other factors such as diabetes, lung disease, depression, neoplasia, and others should also be considered when deciding on diagnosis and treatment [5].

If BP values ≥ 140/90 mmHg are repeatedly found in geriatric patients (previous recommendations were more conservative mentioning values 160/100 mmHg but findings from recent studies confirm that older patients also benefit from the treatment of grade 1 hypertension), their treatment should start and aim to lower BP to values below 140/90 mmHg following the latest recommendations (patients aged 65+ years should have systolic blood pressure (SBP) values between 130 and 140 mmHg and diastolic blood pressure (DBP) values below 80 mmHg [6]). The correct compensation can be then checked using ambulatory blood pressure monitoring (ABPM), with arbitrarily set values for 24-h, daytime and nighttime averages regardless of age. Recently, there have been discussions as to whether the treatment of hypertension in elderly and frail geriatric patients is effective and cost-effective despite their advanced age. Many placebo-controlled studies (e.g., SHEP [7], SYST-EUR [8], Beckett et al. [9]) show that the treatment of hypertension significantly reduces the risk of CV events in the elderly. The conclusions of the HYVET study [10] demonstrate that antihypertensive treatment is effective and positively affects the prognosis in patients aged ≥80 years with a significant reduction in the incidence of heart failure, fatal stroke, and overall mortality. Also, Williamson et al. [11] studying patients aged 75 years or older reported that treating to an SBP target <120 mmHg (compared with an SBP target <140 mmHg) resulted in significantly lower rates of fatal and nonfatal major CV events and death from any cause. However, PARTAGE [12] study focusing on frail patients (>80 years) shows a significant interaction between low SBP (<130 mmHg) and treatment with 2 or more BP-lowering agents, resulting in a higher risk of mortality. Excessive reduction of the BP in the frail geriatric patients, especially in individuals with CV disease, might be detrimental, probably due to a hypoperfusion of target organs [12]. Current 2018 ESC/ESH Guidelines states that the intended target SBP for all patients elder than 65 years is 130–139 mmHg if achievable and tolerated as any BP lowering towards this target is likely to be beneficial. However, it is recommended to avoid treated SBP values of <130 mmHg [6].

ABPM provides more BP measurements, reflects the duration and efficacy of antihypertensive medication over a 24-h period, provides information on nocturnal BP, and more reproducible information than occasional measurements in the office. Thus, thanks to ABPM, we can detect masked hypertension (MH), which is defined as normal BP in the clinic or office (<140/90 mmHg) but increased BP outside the office and medical centers (ambulatory daytime BP or home BP > 135/85 mmHg) [10]. Masked hypertension occurs in 10–20% [13] or even up to 30% of patients [6]. Initial markers of MH are considered to be nocturnal hypertension and non-dipping [14]. Yano and Bakris [15] suggested that MH can be classified based on masked daytime versus masked nighttime patterns. One group of patients may show selective daytime MH when they are exposed to job strain, mental stress, smoking, excessive drinking, or poor exercise tolerance. On the other hand, nocturnal MH can be seen in patients with sleep deprivation, obstructive sleep apnea, metabolic syndrome, diabetes, or chronic kidney disease. In many patients, both can be observed.

Studies show that patients with MH are more likely to experience CV events as a result of high BP and thus MH pose a higher risk than white coat hypertension also in elderly patients [16]. These patients experience more severe damage to target organs as well. Masked hypertension often occurs in patients with chronic kidney disease and leads to faster progression of renal failure [17]. Undiagnosed and untreated MH and treated but uncontrolled MH are two significant high-risk factors impacting public health [14].

Franklin et al. [14] mention that the risk of MH is higher in smokers and individuals who consume alcohol excessively, in people experiencing stress (when measured in the office they may show normal BP values, BP increases in stressful situations), in individuals with sleep deprivation or sleep apnea, in elderly men due to decreased baroreceptor sensitivity and increased BP variability, in obese individuals, patients with diabetes or longer duration of hypertension. Each of these characteristics increases the risk of cardiovascular diseases (CVD).

The objective of this study is to determine the prevalence of masked uncontrolled hypertension (MUH) in frail geriatric patients with arterial hypertension and thus show the role of ABPM in group of frail geriatric patients.

## 2. Materials and Methods

We studied consecutively a sample of 118 geriatric patients treated for arterial hypertension (42 men), mean age 84.2 ± 4.4 years, who had office BP < 140/90 mmHg. Patients were seated comfortably in a quiet environment for 5 min before the beginning of BP measurements. Three BP measurements were recorded, 1–2 min apart. We performed additional measurement if the first two readings differed by >10 mmHg. BP was recorded as the average of the last two BP readings. We used standard bladder cuff and measured the BP in a seated position in all patients following the ESC/ESH guidelines [6]. The measurement was taken by a regularly calibrated standard sphygmomanometer. Furthermore, patients underwent clinical examinations, including heart rate and electrocardiography (ECG) measurements. We assessed frailty in patients using the frailty index because it is complex and precise (in comparison to, e.g., the Barthel test). Frailty index takes into account both physical and psychosocial aspects of frailty and also cognitive functions. The frailty index was calculated as the number of deficits in a patient divided by all considered deficits (70 clinical deficits from the CSHA clinical assessment including the presence and severity of current diseases, ability in ADLs and physical signs from clinical and neurologic exams) [18]. To indicate severity, each deficit not restricted by its nature to two values (i.e., 0 or 1 for absence or presence, respectively) was assigned three (0, 0.5 or 1) or four values (0, 0.33, 0.67 or 1.0), as appropriate. The frailty index ranges from 0.00 to 1.00, a higher value indicates a frailer (worse) status. The Mobil-O-Graph^®^ NG was used for ABPM performed at patient’s habitation in accordance with the currently valid ESC/ESH guidelines [6]. BP was measured every 20 min during the daytime (6 a.m.–10 p.m.) and every 30 min at nighttime (10 p.m.–6 a.m.). Target BP values were the following: mean BP (MBP) 130/80 mmHg during 24 h; MBP 135/85 mmHg during the daytime and MBP 120/70 mmHg during the nighttime [6,15]. ABPM was considered successful when the record provided a minimum of 20 valid daytime and 7 nighttime measurements, and at least 70% of the expected 24-h readings were valid in accordance with ESC/ESH guidelines [6].

As MH were considered SBP ≥ 130 and/or DBP ≥ 80 mmHg during 24 h; SBP ≥ 135 and/or DBP ≥ 85 mmHg during the daytime and SBP ≥ 120 and/or DBP ≥ 70 mmHg during the nighttime.

Isolated systolic hypertension was defined as SBP ≥ 140 and DBP < 90 mmHg.

Patients with malignant disease, patients using nonsteroidal anti-inflammatory drugs, patients with diagnosed secondary hypertension, and patients whose life expectancy is due to malignant or chronic disease less than 1 year were not included.

## 3. Results

Basic clinical parameters are shown in Table 1.

Average office BP and ABPM BP results are shown in Table 2.

**Figure 1:**
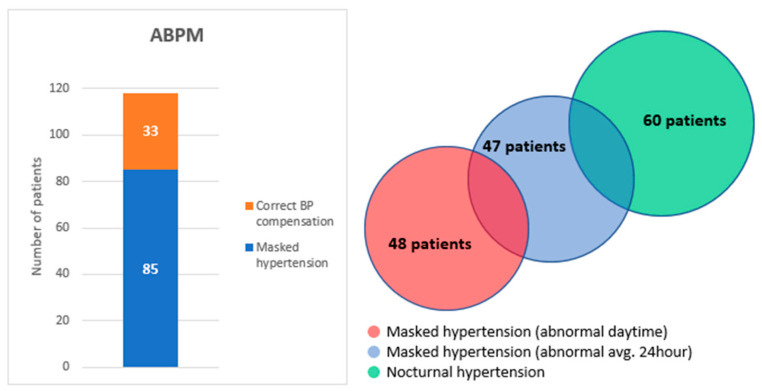
Correct BP compensation and prevalence of masked/nocturnal hypertension (*n* = 118). BP = blood pressure, ABPM = ambulatory blood pressure monitoring.

Distribution of number and percentage of patients based on various aspects circadian rhythm are shown in Table 3 and graphically displayed in Figure 1. 

Antihypertensive therapy in patients included angiotensin-converting enzyme (ACE) inhibitors, angiotensin receptor blockers (ARBs), beta blockers, calcium channel blockers (CCBs), centrally acting antihypertensives and diuretics. Distribution of antihypertensive medication can be seen in Table 4.

Patients had the following medical history: 48 patients (41%) had ischemic heart disease, 26 patients (22%) had ischemic disease of the lower extremities, 10 patients (8%) had stroke/transient ischemic attack (TIA), 37 patients (31%) had diabetes mellitus and prediabetes (IGT) and 352 patients (30%) had atrial fibrillation.

ECG detected silent myocardial infarction in 8 patients, arrhythmia (mostly the atrial fibrillation) appeared in 38 patients. There was no prevalence in group of patients with MUH.

All 118 patients participated until the end of this study and are still being monitored. ABPM revealed MUH in 85 patients (72%): 47 patients (40%) during 24 h, in 48 patients (41%) during the daytime and only nocturnal hypertension was observed even in 60 patients (51%). 

An exaggerated morning surge in BP occurred in 20 (17%) individuals. Isolated systolic hypertension was observed in 79 (67%) patients.

## 4. Discussion

Prevalence of MUH in fragile geriatric patients has not received much attention yet as evidenced by the lack of available research data. In our study, all patients were normotensive when measured BP in the office and were therefore expected to have well-controlled hypertension. However, ABPM surprisingly detected masked hypertension in almost ¾ of the patients: 40% showed abnormal BP average during 24 h, 41% daytime and 51% nocturnal MH. Our findings are similar to findings of Cacciolati et al. [19], who based on daytime HBPM reported MH in 41% of patients aged ≥75 years with normal BP values in the office. They report that MH increased sharply with the level of office SBP (from 22% in patients with SBP < 120 mmHg to 48% in patients with SBP ≥ 130 mmHg). Other major risk factors are male gender, diabetes, age 80+, antihypertensive use, and e.g., BMI ≥ 25 kg/m^2^. The high frequency of MH in the elderly may be partly explained by the aging-related decrease of the baroreflex and the increased BP variability [19]. Ohkubo et al. [20] mention that MH is also associated with increased CV risk in women and treated patients, regardless of numbers of risk factors or CV complications. Even in patients with low CV risk, MH leads to a significantly higher risk of stroke and CV mortality [20,21]. Also, Bobrie et al. [22] inform that MUH is associated with an increased risk of CVD even in individuals treated with antihypertensives. Franklin et al. [23] reported MH in 44.5% of untreated middle-aged and elderly patients, in another study [24], MH prevalence with untreated was 18.8% but 30.5% in treated patients. Moreover, normotensive diabetic patients had a prevalence of MH of 29.3% when untreated and 42.5% with treatment. Thus, prevalence of MH is not only influenced by diabetes mellitus and other high-risk disease but also by antihypertensive treatment since treatment aimed at normalizing conventional office BP will increase the percentage of patients with MUH [25] when ABPM is not involved in the treatment management.

According to Pierdomenico et al. [16], elderly patients with MUH had a significantly higher CV risk after treatment with various covariates than patients with controlled hypertension.

Nocturnal hypertension was present in 60 (51%) patients. Available data show that nocturnal hypertension causes a significant increase in CVD and CV morbidity and mortality. Patients with coexistence of blunted SBP decline have the worst risk profile [4]. Agarwal et al. [26] reported that almost 60% of patients treated for chronic kidney disease had MUH, which was diagnosed exclusively 24% of the time in nocturnal ABPM. Nocturnal hypertension leads to faster chronic kidney disease progression [27]. ABPM is thus very beneficial, especially to these patients, for its contribution to the diagnosis and treatment of nocturnal BP, which is almost always elevated in patients with moderate to severe renal impairment [28].

Higher nocturnal BP and non-dipping is also associated with slower walking speed [4,29]. Decreased mobility during the day in frail seniors can lead to a decrease in physical activity, which can in turn affect daytime BP and lead to the disappearance of nocturnal BP dipping [30]. As shown by J-SHIPP study [31], nocturnal BP is associated with mild cognitive dysfunction in the elderly. According to Garcia et al. [5], along with high nocturnal SBP and non-dipping, increased BP variability is also considered an important determinant of cognitive impairment. Furthermore, 24-h SBP has been shown to be an independent factor for brain atrophy in the elderly [32]. Even though association of BP and cognitive performance is diversified in people elder than 65 years, with more studies showing the worse cognitive function with high or low BP, (in contrast to younger individuals in which high BP is linked to cognitive impairment) [33], strict control of BP, including nighttime, is recommended as it can have a neuroprotective effect and prevent the occurrence of dementia. It is suggested that ABPM may help with an early diagnosis of mild cognitive impairment (MCI) since CV and neuro-cognitive systems operate in relation and suboptimal BP can be considered an early biomarker of cognitive impairment [33]. Prescription of evening antihypertensives is often necessary to improve the prognosis in patients with nocturnal hypertension. Moreover, antihypertensive therapy might also reduce the risk of developing cognitive impairment as suggested by different studies [33].

An exaggerated morning surge in BP was observed in 20 (17%) patients. Morning rise in BP is a common physiological process, but an exaggerated surge in BP in the morning hours after waking and getting up is a serious risk factor, especially for frail geriatric patients. Abnormal morning surge is when SBP values are higher than 50 mmHg and/or DPB values are higher than 22 mmHg between 6 a.m. and 10 a.m. compared to the nocturnal MBP. Exaggerated morning BP may be a sign of insufficient choice of antihypertensive regimen, e.g., administration of short-acting or medium-acting drugs, undermedication or none/insufficient use of combination therapy.

A very frequent phenomenon in our target group detected in 79 (67%) patients was also isolated systolic hypertension, i.e., SBP ≥ 140 mmHg and DBP < 90 mmHg, which increases with age. These findings are similar to results of NHANES III [34] study where isolated systolic hypertension occurred in 87% of individuals in the group of patients 60+ years. Isolated systolic hypertension with increased pulse BP occurs due to the loss of arterial elasticity in geriatric patients. Decreased arterial compliance and increased pulse pressure are associated with up to 4 times higher risk of myocardial infarction, left ventricular hypertrophy, renal dysfunction, stroke, and CV mortality [35].

The above-mentioned trends show the importance of treatment control using ABPM even in fragile geriatric patients since it provides additional data for treatment management. Although we currently have the means of modern pharmacology and practice effective combination therapy in the patient, our research shows that proper BP compensation is only achieved in some patient because quite often the treatment of hypertension does not involve 24-h monitoring, both at the beginning and after certain periods during the treatment. When the diagnosis and treatment are indicated, BP measurements are often performed only in the office which seems to be insufficient for the adequate treatment. It is very important to know the complex 24 h BP profile of the patient. Despite the unclarity whether using ambulatory BP values to guide therapy in patients with MUH leads to reduction of morbidity and mortality [6], our study shows that ABMP is a great complementary alternative in treatment management and the right dosage of medication. It not only provides more data for better estimation of CV events than clinical BP measurements, offers tailor-made treatment and tailoring therapy to an individual BP profile, and can help us address daily life situations (explain stress-related BP increases, detect an in/sufficient decrease during the sleep, and identify over/or under-treatment), but it is thus currently the most accurate and potentially most cost-effective. Moreover, using ABPM does not cause white coat hypertension in patients. ABPM should be performed with each patient who has an elevated BP ≥ 140/90 mmHg recorded by any measurement method (if ABPM is rejected or not tolerated, home BP measurement is recommended). Once the treatment is initiated, we recommend that ABPM is repeated after a few weeks to determine whether the treatment is effective and the decrease in BP is adequate. As an example, in RAMBLER study [36], treatment in 38% of patients was adapted thanks to ABPM, 32% of patients initiated new treatment, and 14% of untreated patients with elevated office BP who were candidates for drug treatment did not initiate treatment because ABPM values were normal.

It should be noted that treatment aiming at normalization of conventional BP in the office and ignoring out-of-office BP measurements may lead to an increase in the percentage of patients with MUH. ABPM is a key tool for a correct management and prognosis in this subpopulation [21,25]. If individuals with MH are not detected, they will remain untreated or insufficiently controlled and may experience CV complications and target organ damage. The quality of life of patients will thus decrease and unnecessary costs for medical care will increase. Despite initial expenditures, ABPM seems to be ultimately cost-effective due to improved BP control, reduction of CV consequences of hypertension, and decreased costs of subsequent treatment. At the same time, the use of ABPM would determine the true level of BP and improve currently “poor” control level of hypertension.

## 5. Conclusions

The results of our study show that for the appropriate treatment setting, it is important to perform 24-h BP measurements even in frail geriatric hypertensive patients with normotensive office BP values. High incidence of MUH in our group manifests the role of ABPM, which is key for management and adequate treatment of frail geriatric patients, also because it detects other phenomena such as exaggerated morning surge and isolated systolic hypertension. As suggested by other studies, their detection and treatment leads to decrease in the incidence of cardiovascular events. Thus, we expect that it also might reduce the financial burden of the society. The further research is needed to confirm the cost-effectiveness of this approach.

## Figures and Tables

**Table 1:** Basic clinical parameters.

Basic Clinical Parameters Sample (*n* = 118) Mean ± SD		
Gender	Male (*n* = 42)	Female (*n* = 76)
Patients	42	76
Age	81.6 ± 4.4	87.1 ± 6.2
Weight (kg)	74.2 ± 17.2	70.2 ± 15.8
Height (cm)	178.9 ± 9.1	161.1 ± 9.1
Body mass index	24.3 ± 5.4	22.1 ± 4.4
Frailty Index (points)	0.6 ± 0.2	0.5 ± 0.2

**Table 2:** Average BP results.

BP Measurement	Time Period	Systolic MBP (mmHg)	Diastolic MBP (mmHg)
(*n* = 118) Mean ± SD			
Office BP			
		126.1 ± 8.1	74.9 ± 6.9
ABPM	24 h	124.3 ± 29.8	72.8 ± 13.9
	Daytime	125.9 ± 30.2	74.6 ± 12.9
	Nighttime	124.1 ± 32.1	70.9 ± 15.1

BP = blood pressure, MBP = mean blood pressure, ABPM = ambulatory blood pressure monitoring.

**Table 3:** Correct BP compensation and prevalence of masked/nocturnal hypertension (*n* = 118).

	*N*	%
(*n* = 118)		
Correct office BP compensation (target values)	118	100
Correct ABPM compensation (target values):	33	28
24 h systolic + diastolic BP	33	28
24 h systolic BP	33	28
24 h diastolic BP	60	51
Daytime systolic + diastolic BP	33	28
Daytime systolic BP	33	28
Daytime diastolic BP	49	58
Night systolic + diastolic BP	8	7
Night systolic BP	15	13
Night diastolic BP	35	30
Masked hypertension:	85	72
Masked hypertension (abnormal avg. 24 h)	47	40
Masked hypertension (abnormal daytime)	48	41
Nocturnal hypertension	60	51

**Table 4:** Antihypertensive medication.

	*N*	%
ACE inhibitors	67	57
Angiotensin receptor blockers (ARBs)	39	33
Calcium channel blockers (CCBs)	62	53
Diuretics	43	36
Beta blockers	38	32
Centrally acting antihypertensives	19	16
Monotherapy	27	23
Dual therapy	32	27
Triple therapy	59	50

## Data Availability

The corresponding authors can provide datasets, which were collected, used and analyzed in this study, on a reasonable request.
